# Pervasive Divergence in Protein Thermostability is Mediated by Both Structural Changes and Cellular Environments

**DOI:** 10.1093/molbev/msaf137

**Published:** 2025-06-06

**Authors:** Nilima Walunjkar, Timothy Y Lai, Nasima Akhter, James H Miller, John Q Bettinger, Erin Marcus, Eric M Phizicky, Sina Ghaemmaghami, Justin C Fay

**Affiliations:** Department of Biology, University of Rochester, Rochester, NY 14610, USA; Department of Biology, University of Rochester, Rochester, NY 14610, USA; Department of Biology, University of Rochester, Rochester, NY 14610, USA; Department of Biology, University of Rochester, Rochester, NY 14610, USA; Department of Biology, University of Rochester, Rochester, NY 14610, USA; Department of Biochemistry and Biophysics, Center for RNA Biology, University of Rochester School of Medicine, Rochester, NY 14610, USA; Department of Biochemistry and Biophysics, Center for RNA Biology, University of Rochester School of Medicine, Rochester, NY 14610, USA; Department of Biology, University of Rochester, Rochester, NY 14610, USA; Department of Biology, University of Rochester, Rochester, NY 14610, USA

**Keywords:** protein evolution, thermotolerance, evolutionary constraints

## Abstract

Temperature is a universal environmental constraint and organisms have evolved diverse mechanisms of thermotolerance. A central feature of thermophiles relative to mesophiles is a universal shift in protein stability, implying that it is a major constituent of thermotolerance. However, organisms have also evolved extensive buffering systems, such as those that disaggregate and refold denatured proteins and enable survival of heat shock. Here, we show that both cellular and protein structural changes contribute to divergence in protein thermostability between two closely related *Saccharomyces* species that differ by 8 °C in their thermotolerance. Using thermal proteomic profiling we find that 85% of *S. cerevisiae* proteins are more stable than their *S. uvarum* homologs and there is a 1.6 °C shift in average protein melting temperature. In an interspecific hybrid of the two species, *S. cerevisiae* proteins retain their thermostability, while the thermostability of their *S. uvarum* homologs is enhanced, indicating that cellular context contributes to protein stability differences. By purifying orthologous proteins, we show that amino acid substitutions underlie melting temperature differences for two proteins, Guk1 and Aha1. Amino acid substitutions are also computationally predicted to contribute to stability differences for most of the proteome. Our results imply that widespread changes in protein thermostability accompany the evolution of thermotolerance between closely related species.

## Introduction

Microorganisms vary widely in the temperatures they experience and their thermal limits. Protein stability is a key component of thermotolerance as heat causes protein unfolding, aggregation, and loss of fitness. Consequently, there are diverse mechanisms for achieving protein homeostasis at different temperatures. For example, the heat shock response is a near-universal response to temperature-induced protein denaturation and is tuned to an organism's upper thermal limit (Parsell and Lindquist 1993; Feder and Hofmann 1999; Riback et al. 2017; Iserman et al. 2020; Keyport Kik et al. 2024). Additionally, the proteomes of thermophiles are exceptionally robust to temperature-induced aggregation (Sterner and Liebl 2001; Leuenberger et al. 2017; Jarzab et al. 2020). Given the frequent and sometimes rapid temperature shifts due to climate change, the evolution of protein stability is an important but complex variable relevant to an organism's thermal tolerance.

Protein stability is influenced by both protein structure and the cellular environment. Most amino acid substitutions destabilize proteins or make it more difficult for them to fold into their native conformations (DePristo et al. 2005; Tokuriki et al. 2007; Klesmith et al. 2017). However, both natural and experimental evolution show that proteins can attain higher thermostability without compromising their activity (Giver et al. 1998; Rahban et al. 2022). The cellular environment can impact protein stability through pH (Whitten et al. 2005), ligand or protein interactions (Savitski et al. 2014; Tan et al. 2018), post-translational modifications (Shental-Bechor and Levy 2008), thermoprotectant molecules (Jain and Roy 2009), and protein chaperones (Mogk et al. 2019). Because most proteins are marginally stable due to the small changes in free energy (Δ*G*) between folded and unfolded states (Taverna and Goldstein 2002), both structural changes and cellular factors may continuously evolve with the temperatures experienced by an organism (Somero 1995; Tokuriki and Tawfik 2009b).

The numerous ways in which stability can be modulated provide multiple evolutionary paths to thermal adaptation. Cellular chaperones and thermoprotectants can yield immediate stabilization of proteins but may be limited to small, temporary, or partial stabilization of the proteome (Tokuriki and Tawfik 2009a; Wyganowski et al. 2013). Structural stabilization of proteins can be broadly achieved through amino acid substitutions, but may be limited by the time and coordination of changes across the proteome. While thermal adaptations have been documented for both paths (Rudolph et al. 2010; Fields et al. 2015; Caspeta et al. 2016; Pinney et al. 2021), experimental evolution in multiple species has resulted in limited increases in upper thermal limits (Mongold et al. 1999; Rudolph et al. 2010; Caspeta et al. 2014; Geerts et al. 2015; Huang et al. 2018; Sasaki and Dam 2021; Longan and Fay 2023). This indicates that protein stability might constrain the rate of temperature adaptation and raises the possibility that proteome-wide stabilization may be required for natural systems to attain systemic increases in thermotolerance.

A major challenge to understanding divergence in protein stability between species is disentangling cellular contributions to stability from those that are structurally encoded by a protein's amino acid sequence. Although changes in stability have been characterized through the purification of individual proteins (Razvi and Scholtz 2006; Fields et al. 2015), proteomic measures of melting temperature divergence between thermophiles and mesophiles do not always distinguish between structural and cellular contributions. In addition to chaperones and thermoprotectants, the cell environment can influence protein stability through post-translational modifications (Shental-Bechor and Levy 2008), the presence of binding partners (Tan et al. 2018), macromolecular crowding, or other constituents of the cell. Proteomic studies have also shown that protein melting temperatures are somewhat dependent on the cell type and environment (Becher et al. 2018; Mateus et al. 2018; Tan et al. 2018) and that melting temperatures of proteins from mesophilic species are not closely tied to their optimal growth temperature (Engqvist 2018; Jarzab et al. 2020). This has made it difficult to understand the extent to which protein stability co-evolves with an organism's thermal niche.

**Fig. 1.**
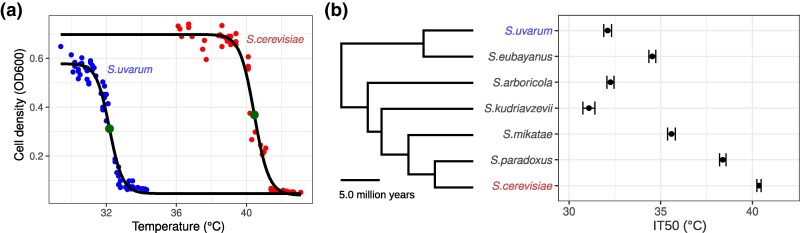
Divergence in thermotolerance among the *Saccharomyces* species. a) Thermotolerance was measured by the temperature at which growth yield (OD600) was reduced by 50% (IT50, large dots), based on treatments using a gradient thermocycler. b) *Saccharomyces* species IT50 with whiskers showing 95% confidence intervals. Species tree and divergence times were obtained from (Shen et al. (2018).

In this study, we characterize proteome stabilities of two thermally diverged *Saccharomyces* species, *S. cerevisiae* and *S. uvarum*. These two species have diverged for 16 million years (Shen et al. 2018) and have evolved an 8 °C difference in their thermal growth limits (Fig. 1), resulting in substantial temperature-dependent fitness differences (Salvadó et al. 2011) and their differential use in low versus higher temperature fermentations (Belloch et al. 2008; Querol and Bond 2009). The two species can also produce viable hybrids, enabling protein stability to be measured in the same cellular environment. Our results provide direct evidence that both structural and cellular changes contribute to widespread changes in protein thermal stability in yeast and imply that protein stability co-evolves with thermotolerance between closely related species.

## Results

### *S. cerevisiae* Proteins Are More Thermostable Than Their *S. uvarum* Orthologs

To investigate divergence in protein stability between *S. cerevisiae* and *S. uvarum,* we used thermal proteomic profiling to measure protein melting temperatures (Savitski et al. 2014). Cell extracts were subjected to temperature treatments and the soluble fraction of proteins was quantified by LC-MS/MS. We estimated the temperature at which the levels of each protein in the soluble fraction were reduced by half and obtained melting temperatures (Tm) for 1,330 (replicate 1) and 914 (replicate 2) orthologous protein pairs (supplementary table S1, Supplementary Material online).

Previously, *S. uvarum* hexokinases were shown to have lower thermal stability compared to *S. cerevisiae* but paralogous hexokinases weren't distinguished (Gonçalves et al. 2011). In our proteomic data, hexokinases (Hxk1, Hxk2, Glk1, and Emi2) from *S. cerevisiae* show higher stability than their *S. uvarum* orthologs (supplementary table S2, Supplementary Material online, supplementary fig. S1, Supplementary Material online). As an additional assessment of stability differences, we measured enzyme activities of malate dehydrogenase (encoded by *MDH1*, *MDH2* and *MDH3*) and glutathione reductase (*GLR1*) following heat treatment of cell extracts. Consistent with our proteomics data (supplementary table S2, Supplementary Material online, supplementary fig. S1, Supplementary Material online), *S. cerevisiae* activity was more resistant to temperature than *S. uvarum* (supplementary fig. S2, Supplementary Material online).

**Fig. 2.**
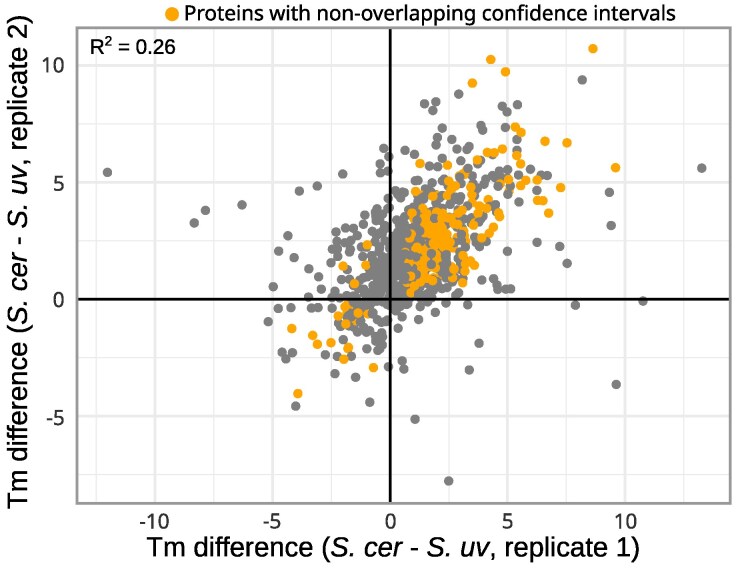
*S. cerevisiae* proteins have higher melting temperatures than *S. uvarum*. Melting temperature (Tm) differences between *S. cerevisiae* and *S. uvarum* for replicate 1 versus replicate 2. *R*^2^ is from the Pearson correlation. Orange points show proteins with melting temperature confidence intervals that did not overlap between species in both replicates (*n* = 206).

Across 827 proteins with data in both species, *S. cerevisiae* proteins on average had a melting temperature 1.6 °C higher than those of *S. uvarum* proteins, and 85% of *S. cerevisiae* proteins were more thermostable than their *S. uvarum* orthologs (Binomial *P* < 0.001, Fig. 2, supplementary table S3, Supplementary Material online, supplementary table S4, Supplementary Material online). In comparison, the average melting temperature difference between species' replicates was 0.63 (supplementary table S5, Supplementary Material online, supplementary fig. S3, Supplementary Material online) similar to a previous study (Jarzab et al. 2020). For proteins with melting temperatures that had non-overlapping confidence intervals between species (*n* = 206), 90% of *S. cerevisiae* proteins were more stable than *S. uvarum* (Fig. 2, supplementary table S3, Supplementary Material online). The large fraction of proteins with higher melting temperatures in *S. cerevisiae* indicates uniform divergence in proteome stability. However, mitochondria and membrane-associated proteins were enriched for higher melting temperatures in *S. cerevisiae* whereas ribosomal proteins were enriched for higher melting temperatures in *S. uvarum* (supplementary table S6, Supplementary Material online). Larger melting temperature differences were associated with lower protein abundance and higher melting temperatures (supplementary table S7, Supplementary Material online). However, using protein abundance as a covariate did not significantly change the average melting temperature difference between species (1.56 vs. 1.55, *P* = 0.09).

### Proteome Stability Depends on the Cellular Environment

**Fig. 3.**
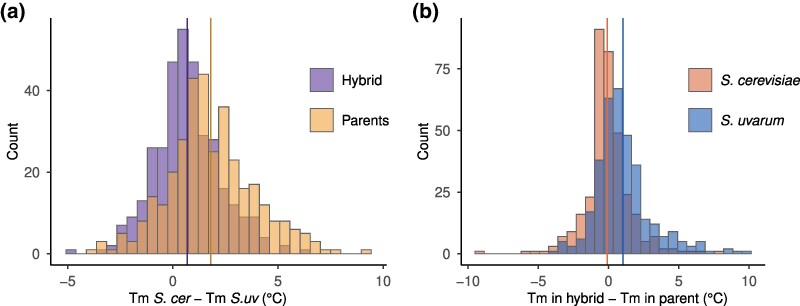
Protein melting temperatures in the hybrid compared to the parental environments. a) Histogram of melting temperature (Tm) differences between orthologs in the hybrid and in their parental species context. b) *S. uvarum* proteins are stabilized in the hybrid environment. The histogram shows the distribution of differences between the melting temperatures measured in the hybrid and the respective parental species for both *S. cerevisiae* and *S. uvarum* proteins. Vertical lines show the average.

Protein stabilities can depend on the cellular environment, due to post-translational modifications, such as glycosylation (Shental-Bechor and Levy 2008), or the stability of other proteins in the same complex (Tan et al. 2018). To control for the cellular environment, we also measured the melting temperatures of proteins from an *S. cerevisiae* × *S. uvarum* hybrid and obtained species-specific measurements of orthologous protein pairs using species-specific peptides. In the hybrid, 67.4% of detected *S. cerevisiae* proteins were more stable than their *S. uvarum* orthologs (supplementary table S3, Supplementary Material online), and when combined with the parental data 87.7% of *S. cerevisiae* proteins were consistently more stable than their *S. uvarum* ortholog across both parental replicates and the hybrid (Binomial *P* < 2.2e−16, *n* = 204, supplementary table S3, Supplementary Material online). However, melting temperature differences between orthologs were significantly smaller in the hybrid (0.68 °C) compared to the same proteins in the parents (1.80 °C) (Fig. 3a, supplementary table S5, Supplementary Material online, Wilcoxon signed rank test, *P* < 2.2e−16), indicating that other factors besides a protein's sequence contribute to the observed melting temperature differences between species.

We tested whether *S. cerevisiae* proteins were destabilized or *S. uvarum* proteins were stabilized in the interspecific hybrid. We found no consistent difference between the hybrid and parental stability of *S. cerevisiae* proteins, but *S. uvarum* proteins were significantly more stable in the hybrid compared with their parental cellular environment (Fig. 3b, paired *t*-test, *P* < 2.2e−16). Given the stabilization of *S. uvarum* proteins in the hybrid, we tested whether hybrid stabilized proteins were associated with glycosylation, membranes, protein complexes, protein–protein interactions, the ribosome, or the mitochondria. We found that membrane proteins were more stable in the hybrid (supplementary table S8, Supplementary Material online), but this was true for both *S. uvarum* (OR = 4.31, Fisher's exact test *P* = 0.0007) and *S. cerevisiae* proteins (OR = 2.78, Fisher's exact test, *P* = 0.0013). There was no significant difference in the membrane enrichment of *S. uvarum* compared to *S. cerevisiae* proteins (Logistic regression, *P* = 0.46). For *S. uvarum*, ribosomal proteins and proteins with more protein–protein interactions were destabilized in the hybrid compared to the parental replicates, but *S. cerevisiae* proteins showed no differences between the two contexts. Thus, the stabilization of *S. uvarum* proteins is not uniquely concentrated in any of the groups of proteins tested.

### Guk1 and Aha1 Stability Differences Are Structurally Encoded

**Fig. 4.**
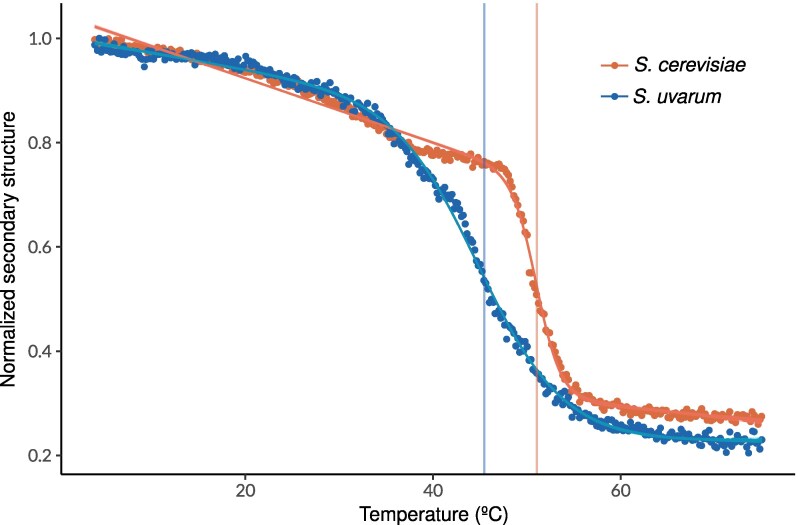
Divergence in Guk1 thermal stability. Circular dichroism was used to measure the thermal loss of secondary structure for *S. cerevisiae* and *S. uvarum* Guk1. Secondary structure was measured by mean residue molar ellipticity (deg cm^2^ dmol^−1^ per residue) at 222 nm as a function of temperature (°C) and then scaled by the minimum value to get normalized secondary structure. Data points represent experimental measurements, while the fitted line corresponds to the model's predictions.

Many proteins exhibit consistent stability differences between the parents and between parental alleles within the hybrid, suggesting that they are structurally encoded by amino acid differences between species. To assess divergence in protein stability due to sequence alone, we selected two proteins (Guk1 and Aha1) and cloned and purified both from *S. cerevisiae* and *S. uvarum*. Guk1 and Aha1 were chosen as they are both small proteins with large and consistent melting temperature differences (supplementary table S2, Supplementary Material online). For each protein, we used circular dichroism spectrometry to characterize their in-vitro melting curves by tracking ellipticity as a function of temperature. *S. cerevisiae* Guk1 had a 5.6 °C higher melting temperature than that of *S. uvarum* (Fig. 4, supplementary fig. S4, Supplementary Material online), demonstrating that the 21 amino acid substitutions have together resulted in the divergence of protein stability. For Aha1, the *S. cerevisiae* protein was also more thermostable than that of *S. uvarum* due to 54 amino acid differences and an insertion/deletion (supplementary fig. S4e, Supplementary Material online, Tm difference of 9.1 °C). We also measured Guk1 activity following thermal treatments of purified protein and confirmed a loss of activity consistent with the protein's melting temperature (supplementary fig. S2, Supplementary Material online).

### Divergence in Guk1 Protein Thermostability Does Not Affect Fitness in *S. Cerevisiae*

Guanylate kinase (Guk1) is an essential protein that catalyzes the conversion of guanosine monophosphate (GMP) to guanosine diphosphate (GDP). To test if Guk1 stability differences between *S. cerevisiae* and *S. uvarum* can cause temperature-dependent growth differences, we replaced the endogenous *S. cerevisiae GUK1* with the less stable *S. uvarum* allele under the *S. cerevisiae* promoter. There were no growth differences under various conditions and temperatures, or upon heat shock at 50 °C (supplementary fig. S5, Supplementary Material online). An allele replacement in *S. uvarum* also showed no change in thermotolerance (supplementary fig. S6, Supplementary Material online). These results indicate that Guk1 alleles are functionally equivalent at these temperatures or that any differences are not large enough to generate detectable fitness effects.

### Predictable Differences in Protein Stability

If the higher melting temperatures of *S. cerevisiae* proteins are structurally encoded by amino acid differences between species, then the replacement of *S. cerevisiae* amino acids with those present in *S. uvarum* should destabilize the protein. To assess the impact of amino acid substitutions on stability, we generated AlphaFold2 structures for *S. cerevisiae* and *S. uvarum* proteins (Jumper et al. 2021 ; Mirdita et al. 2022) and predicted the difference in Δ*G* (ΔΔ*G*) using the structure-based antisymmetric convolutional differential concatenated neural network (ACDC-NN) method (Benevenuta et al. 2021). We also generated predictions for substitutions of *S. cerevisiae* amino acids into predicted *S. uvarum* protein structures to avoid any reference structure bias.

**Fig. 5.**
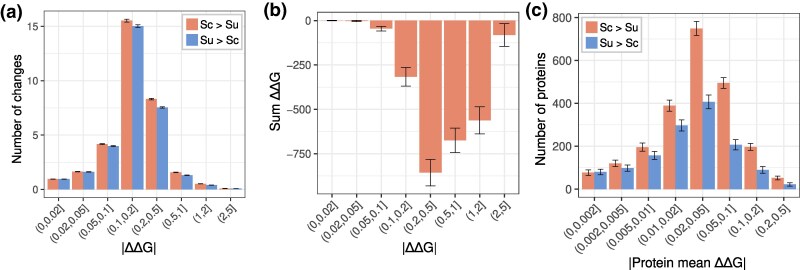
Distribution of protein stability differences between species. a) Distribution of the average number of amino acid changes per protein where the *S. cerevisiae* allele is more stable than *S. uvarum* (Sc > Su) or vice versa (Su > Sc), binned by the absolute value of ΔΔ*G*. Whiskers show a 95% confidence interval based on the standard error. b) Sum of positive ΔΔ*G* and negative ΔΔ*G* values, binned by absolute value of ΔΔ*G*. Whiskers show bootstrap estimates of the standard error. A negative sum indicates *S. uvarum* alleles are cumulatively destabilizing. c) Histogram of average protein stability differences for proteins with higher stability in *S. cerevisiae* or *S. uvarum*. Whiskers show the binomial 95% confidence intervals.

The average protein has 64 amino acid differences between the two species, corresponding to 87% identity, and 1.9 more stabilizing amino acids in *S. cerevisiae* compared to *S. uvarum* (Fig. 5a). Over the entire proteome, there is a balance in the accumulation of stabilizing and destabilizing changes with small (|ΔΔ*G*| < 0.1) and large (|ΔΔ*G*| > 2) effects, but an accumulation of intermediate effects with higher stability in *S. cerevisiae* (Fig. 5b). These stability differences are mostly derived from regions with high confidence in AlphaFold structures and are also found in ACDC-NN-Seq ΔΔ*G* predictions based on sequence alone (supplementary tables S9 and S10, Supplementary Material online). Assessing the combined effects of these changes across a protein is difficult due to epistasis, which tends to occur between sites that are physically close to one another, five residues or five Å (Faure et al. 2024). Assuming no epistasis and using the average ΔΔ*G* of each protein we find that 62.6% of *S. cerevisiae* proteins are predicted to be more stable than their *S. uvarum* orthologs (Fig. 5c, Binomial *P* < 1e−15). Using a metric based on the largest deviations [max + min − mean (Montanucci et al. 2019)], 56.5% of *S. cerevisiae* proteins are predicted to be more stable (Binomial *P* = 3.2e−15). Thus, the average protein ΔΔ*G* value is small when sites are combined by the mean (−0.0156), the largest deviation metric (−0.068), or the sum (−0.700).

Changes in Δ*G* can be correlated with changes in melting temperature depending on how the enthalpy and entropy of unfolding changes with temperature (Becktel and Schellman 1987). However, we found no correlation between protein ΔΔ*G* metrics (mean, sum, or largest deviation) and their difference in melting temperature estimated from thermal proteomic profiling (Spearman's ρ = −0.02 to −0.07, *P* > 0.05). Similarly, average ΔΔ*G* values for Guk1 (0.001) and Aha1 (0.008) were not suggestive of their measured melting temperatures.

## Discussion

Species that inhabit disparate thermal environments exhibit evidence of thermal adaptation based on differences in their induction of the heat shock response and in their protein melting temperatures. Prior studies have refined these observations by identifying specific proteins that are thermal sensors and have diverged between even closely related species (Riback et al. 2017; Iserman et al. 2020; Keyport Kik et al. 2024). Our results demonstrate a proteome-wide shift in thermostability between two thermally diverged mesophilic *Saccharomyces* species. We also show that changes in the cellular environment contribute to protein melting temperature divergence. However, most proteome stability differences persist even in the same cellular environment. By showing that thermostability divergence is structurally encoded for Guk1 and Aha1 and is structurally predictable across the proteome, our results imply that divergence in thermotolerance involves massively concordant changes in protein stability. However, we also find no detectable fitness effects caused by Guk1 divergence. Below, we discuss our results in relation to thermal divergence and adaptation.

### Coordinated Evolution of Protein Melting Temperatures

Most thermophilic and mesophilic organisms have been separated for billions of years and exhibit large differences in protein melting temperatures throughout their proteomes (Razvi and Scholtz 2006; Stetter 2006; Engqvist 2018; Jarzab et al. 2020). We estimate that nearly 85% of proteins in *S. cerevisiae* are more stable than their *S. uvarum* orthologs, implying a similarly massive set of coordinated changes in protein thermostability between relatively closely related yeast species. This estimate excludes proteins with inconsistent differences between replicates but is fairly uniform across protein groups with the exception of ribosomal proteins. The melting temperature differences in the hybrid, while smaller in magnitude, are largely concordant with the parental species differences and indicate that dominant cellular factors from *S. cerevisiae* stabilize *S. uvarum* proteins in the hybrid. This cellular dominance could contribute to the hybrid's ability to grow at high temperatures (37 °C) (Li and Fay 2017).

There are multiple factors that could contribute to *S. cerevisiae*'s stabilizing cellular environment. Glycosylation or other post-translation modifications influence stability (Shental-Bechor and Levy 2008). Stability is also influenced by protein complexes, which are known to be chimeric in the hybrid environment and destabilized in strains with interspecific chromosome substitutions (Dandage et al. 2021; Swamy et al. 2022). We found stabilization of membrane proteins in the hybrid environment, but the stabilizing effects were observed for proteins from both species. Membrane fluidity is influenced by lipid composition, which varies in the *Saccharomyces* species (Redón et al. 2011; Froissard et al. 2015; Ruiz-de-Villa et al. 2022) as well as in response to temperature (Puig-Castellví et al. 2018). Thus, the membrane lipid environment is one mechanism by which membrane protein thermostability could be altered in the hybrid context (Vitrac et al. 2019). However, our results are also limited as they do not address in vivo stability, where the concentration of cellular factors is important. Further work will be needed to identify how cell environments diverge between species, as such changes could play an important role in the evolution of thermotolerance.

### Fitness Consequences of Protein Stability

A central concept from comparative studies is that selection shapes the distribution of protein melting temperatures across species. However, selection on melting temperatures is most likely indirect and caused by selection on protein stability or other biophysical properties at physiological temperatures since most protein melting temperatures greatly exceed an organism's thermal growth limits. Prior studies have shown that divergence in protein melting temperatures can result from changes in its heat capacity and/or enthalpy and is most likely driven by selection to prevent misfolding and maintain protein activity (Bloom et al. 2006; Razvi and Scholtz 2006; Hart et al. 2014). This biophysical model of protein evolution is supported by the observation that most proteins are marginally stable such that most mutations are destabilizing (Taverna and Goldstein 2002; Tokuriki and Tawfik 2009b; Echave and Wilke 2017).

Consistent with the idea that selection acts to maintain levels of protein stability at the temperatures experienced by an organism, we find that amino acid differences between the two species are predicted to stabilize *S. cerevisiae* or destabilize *S. uvarum* proteins. However, we also find no correlation between protein melting temperature differences and the sum of the predicted ΔΔ*G* across a protein. Besides measurement error, the absence of a correlation could indicate that changes in protein stability are not additive or that the temperature of aggregation (Tm) differs from the temperature of unfolding (ΔΔ*G*) in a protein-specific way. Prior studies have shown that non-additive (epistatic) effects among protein substitutions are common and increase as protein homologs diverge (Pokusaeva et al. 2019; Gonzalez Somermeyer et al. 2022). A further complication is that ΔΔ*G* is environment-dependent but ΔΔ*G* prediction methods are trained on empirical data that is not from a uniform environment (Sanavia et al. 2020).

Proteins vary in how changes in stability are related to fitness, with some proteins being more robust to changes in stability than others (Gonzalez Somermeyer et al. 2022). In the case of Guk1, we find no detectable fitness effects caused by placing the thermally sensitive *S. uvarum* allele into an *S. cerevisiae* background. One explanation is that *S. uvarum* Guk1 is less stable at physiological temperatures but protein levels are not sufficiently reduced to generate detectable fitness effects. Prior studies have shown that while null alleles of *GUK1* are recessive lethal (Konrad 1992), temperature-sensitive alleles of *GUK1* are unstable and degraded by the proteasome at restrictive temperatures (Comyn et al. 2016). Thus, even if thermally sensitive *S. uvarum* alleles do not substantially affect fitness at high temperatures, the combined effects of numerous thermosensitive *S. uvarum* proteins could limit thermotolerance via a denaturation catastrophe (Dill et al. 2011).

### Protein Stability as a Mechanism of Thermotolerance

How does protein stability evolve in relation to thermotolerance? Given the preponderance of destabilizing mutations, loss of stability may easily occur as a neutral process when there is no selection for thermostability via exposure to high temperatures. Thus, one possibility is that the ancestor of the *Saccharomyces* had moderate to high thermotolerance which was then lost along with protein stability on each of the lineages leading to thermally sensitive species. Consistent with parallel loss of stability, *S. kudriavzevii* proteins aggregate at a lower temperature in vivo than *S. cerevisiae* proteins (Keyport Kik et al. 2024). However, the results of this study show an adaptive role in tuning a species' heat shock response. Another non-exclusive evolutionary scenario is the gain of thermotolerance along the lineage leading to *S. cerevisiae* (Fig. 1) (Weiss et al. 2018). In the context of increasing thermotolerance, thermostability may impose a significant constraint on the rate of adaptation: a mutation that enhances the stability of one protein may not have much impact on thermotolerance without concordant changes in the rest of the proteome. Thus, stabilization of the entire proteome may be constrained to incrementally occur through many changes of small effect. However, changes in the cell environment, e.g. due to thermoprotectants like trehalose (Felix et al. 1999) or protein chaperones like Aha1 (Lotz et al. 2003), provide one mechanism that could alleviate the constraints of coordinated changes throughout the proteome by generating a temporary buffer that facilitates the evolution of higher thermotolerance.

## Methods

### Species’ Thermotolerance

We measured thermal tolerance by the final density of cultures grown across a range of temperatures for representative strains of seven species (supplementary file S2, A, Supplementary Material online). Overnight rich medium (YPD: 1% yeast extract, 2% peptone and 2% dextrose) cultures were grown to mid-log phase and diluted 1:100 (vol:vol) in fresh medium. Cultures (100 µl) were grown in PCR tubes and incubated in a gradient thermocycler across a range of temperatures for 24 h. For each gradient, temperatures were empirically measured to avoid slight inaccuracies in the machine-estimated gradient. Final concentrations were measured by OD600 in flat bottom plates. The temperature at which growth was inhibited by 50% along with confidence intervals was estimated for each species by fitting a five-parameter least squares regression using the nplr package (v0.1–7) in R (Commo and Bot 2025).

### Thermal Proteome Profiling

#### Lysate Preparation

We characterized proteome thermostabilities of two replicates of the haploid parental strains, *S. cerevisiae* (YJF155) and *S. uvarum* (YJF1449), and one replicate of the interspecies hybrid (YJF4680: YJF155 x YJF1449, supplementary file S2, A, Supplementary Material online). Strains were grown overnight at 25 °C in YPD, resuspended in fresh medium, and harvested during the log phase after 4 h of growth. Cells were combined in equal portions with cells from the overnight culture to capture proteins expressed in both log and stationary phase cells. Cells were lysed by bead beating in lysis buffer (20 mM sodium phosphate, 50 mM NaCl, pH 7.4, and 1 EDTA-free protease inhibitor tablet from ThermoFisher). The lysate was sonicated to shear any DNA present and soluble protein was collected from the supernatant after centrifugation at 18,000 *g* for 20 min at 4 °C.

#### Temperature Treatments

Protein lysates were quantified using the bicinchoninic assay (Pierce BCA) and diluted to 2 µg/µl in lysis buffer. Protein extracts (50 µg aliquots) were incubated at temperatures ranging from 30 °C to 72 °C for 3 min in a thermocycler (supplementary file S2, B, Supplementary Material online). We used 10 temperatures for the parental species [Tandem Mass Tags (TMT 10plex)] and 16 different temperatures for the hybrid (TMT 16plex). Bovine serum albumin (BSA) (1 µg) was added as a standard to each tube, and samples were centrifuged at 18,000 *g* for 20 min at 4 °C to remove aggregated proteins.

#### Desalting and TMT Tagging

The supernatant containing the soluble fraction was applied to Ultracel 10K filters (Milipore Sigma) and washed with urea buffer [8 M urea, 100 mM triethylammonium bicarbonate (TEAB, pH 8.5)] by centrifuging at 14,000 *g* for 15 min. The samples were subsequently washed with TCEP buffer [5 mM TCEP (CAS #51805-45-9), 8 M urea, 100 mM TEAB], iodoacetamide (IAA) buffer (50 mM IAA, 8 M urea, 100 mM TEAB) and 100 mM TEAB. Next, samples were digested with 100 µL of trypsin solution (Thermo Fisher #90058—lyophilized powder diluted 1:50 in 100 mM TEAB) overnight at 37 °C. The digest was collected by centrifugation, dried down using a SpeedVac, and frozen at −80 °C.

Samples were resuspended in 100 µL of 0.1% trifluoroacetic acid (TFA) in LC-MS grade water. We then activated stage tips by two washes with 100 µl of 50/50 acetonitrile (ACN) and 0.1% TFA, and two washes with 0.1% TFA. We then applied the resuspended samples to the stage tips and spun them down. Samples were washed twice with 100 µl of 0.1% TFA. All spin steps were carried out at 2200 *g* for 4 min. Next, we eluted with 100 µl of 50/50 ACN and 0.1% TFA. We again dried down and froze the samples at −80 °C.

Samples were resuspended in 125 µl of 100 mM TEAB. 25 µl of the sample was labeled with 20 µl of isobaric TMT (Thermo Fisher) and resuspended in ACN. Parental replicates 1 and 2 were tagged with a randomized TMT10plex, whereas hybrid samples were tagged with a randomized TMT16plex. Samples were quenched with 5 µl of 5% hydroxylamine for 15 min after incubating at room temperature for 1 h. The 10 (or 16 for hybrid) samples were combined, dried down, and frozen.

#### Fractionation

The combined sample was chemically fractionated into 8 fractions using a stage tip activated with two washes of 50 µl of ACN and two washes of 50 µl of 100 mM ammonium formate (AF). The sample was resuspended in 50 µL of 100 mM AF and applied to the activated stage tip. For the parental replicates, eight elution buffers (E1–E8) and a wash buffer were prepared using varying ratios of acetonitrile and 100 mM ammonium formate (10% ACN to 50% ACN). For the hybrid, we used 16 elution buffers (E1–E16) ranging from 2% ACN to 50% ACN (supplementary file S2, C, Supplementary Material online). After adding the wash buffer to the stage tip, we sequentially elute with 50 µl of the elution buffers (E1–E8 or E1–E16), collecting each flow-through in a new tube. For all these steps, we spun samples at 2200 *g* for 2 min. For the hybrid, we combined fractions E1 and E9, E2 and E10, etc., to end up with 8 fractions. The eight eluted fractions were frozen, dried down in a SpeedVac, resuspended in 0.1% TFA, and processed at the University of Rochester Mass spectrometry core for LC/MS-MS.

#### LC/MS-MS

Peptides were injected onto a homemade 30-cm C18 column with 1.8-um beads (Sepax), with an Easy nLC-1200 HPLC (Thermo Fisher), connected to a Fusion Lumos Tribrid mass spectrometer (Thermo Fisher). Solvent A was 0.1% formic acid in water, while solvent B was 0.1% formic acid in 80% acetonitrile. Ions were introduced to the mass spectrometer using a Nanospray Flex source operating at 2 kV. The gradient began at 3% B and held for 2 min, increased to 10% B over 7 min, increased to 38% B over 94 min, then ramped up to 90% B in 5 min and was held for 3 min, before returning to starting conditions in 2 min and re-equilibrating for 7 min, for a total run time of 120 min. The Fusion Lumos was operated in data-dependent mode, with both MS1 and MS2 scans acquired in the Orbitrap. The cycle time was set to 2 s. Monoisotopic Precursor Selection (MIPS) was set to Peptide. The full scan was collected over a range of 400 to 1500 m/z, with a resolution of 120,000 at m/z of 200, an automatic gain control (AGC) target of 4e5, and a maximum injection time of 50 ms. Peptides with a charge state between 2 and 5 were picked for fragmentation. Precursor ions were fragmented by higher-energy collisional dissociation using a collision energy of 38% and an isolation width of 1.0 m/z. MS2 scans were collected with a resolution of 50,000, a maximum injection time of 105 ms, and an AGC setting of 1e5. Dynamic exclusion was set to 45 s.

### Data Analysis

#### Peptide Processing

MaxQuant (v1.6.17.0) was used to identify peptides from the LC/MS-MS data and map them to reference proteins of *S. cerevisiae* and *S. uvarum* nuclear (Scannell et al. 2011) and mitochondrial genomes (NC_027264 and NC_031512). We used the default parameters for modifications, setting the group-specific parameter type to Reporter ions MS2 and setting the digestion type to Trypsin (see supplementary table S11, Supplementary Material online for parameters). The hybrid LC/MS-MS data was separately mapped to *S. cerevisiae* and *S. uvarum* and a modified MaxQuant xml file was used to handle the TMT16plex data (available at OSF). For the hybrid dataset, peptides that mapped to both *S. cerevisiae* and the *S. uvarum* proteome were removed (43% of detected peptides). Peptide abundance was extracted from MaxQuant evidence files and used as input for MSTherm (v0.4.7), an R package used to analyze thermal proteomic profiling (TPP) data (Volkening 2017). To compare proteins between species we used orthologs previously determined by homology and synteny (Scannell et al. 2011). From 5261 orthogroups, we removed 24 due to highly similar paralogs, large differences in protein length, or small regions of homology compared to overall protein length. *S. cerevisiae* gene names from the Saccharomyces Genome Database (SGD) were used for all orthologous protein pairs.

#### Measuring Protein Melting Temperatures

All three datasets were standardized (to BSA spike-in) and normalized using MSTherm. Proteins with three or more peptide spectra matches (PSM) were retained. To compare the three datasets, the melting temperatures of the hybrid and second parental replicate were normalized to the first parental replicate. This was accomplished by first estimating Tm values for each protein in the datasets separately, then fitting a linear regression between the Tm values in the first parental replicate and the replicate to be normalized. Importantly, each of the three sets included both *S. cerevisiae* and *S. uvarum* melting temperature values, resulting in equivalent adjustments of each. The regression was then used to adjust the hybrid and second parental set of temperature values. Finally, normalized melting temperature estimates were obtained for each dataset using the adjusted temperature values. The temperature vectors pre- and post-normalization are listed in supplementary file S2, B, Supplementary Material online. All melting temperatures were obtained using the four-parameter logistic function in MSTherm.

#### Filtering Criteria

We removed proteins with missing or low-quality melting temperatures after normalization. First, we removed proteins that didn't have orthologs. Second, we removed proteins with r^2^ < 0.8 for the logistic fit of the melting temperature curve. Third, we removed proteins that only had data in one species within a given replicate. For the two parental replicates, this resulted in 827 protein pairs, and for all three (parents + hybrid), this resulted in 344 orthologous protein pairs. The smaller size of the hybrid dataset was caused by fewer proteins with at least three species-specific PSMs.

MSTherm estimates 95% confidence intervals for proteins with at least eight PSMs using bootstrap resampling. There were 462 proteins with confidence intervals in both parental replicates, of which 206 had non-overlapping confidence intervals between species in both replicates. Only 129 proteins had confidence intervals in the hybrid and the intersection of proteins with non-overlapping confidence intervals in all three datasets was 34.

#### Protein Annotations

Proteins were classified based on whether they are essential, membrane-associated, mitochondrial, glycosylated, ribosomal, or part of a protein complex. A manually curated set of 1900 proteins in 616 complexes in yeast was obtained from the Complex Portal (Meldal et al. 2019). Out of the 827 proteins we examined, 310 were in one or more of these complexes. SGD was used to identify membrane-associated proteins (149), mitochondrial proteins (127), and subunits of the ribosome (62), and to classify proteins as essential (246), non-essential (527), or undetermined (54). Using data from Neubert et al. (Neubert et al. 2016) 98 of the 827 proteins were annotated as glycosylated. Aggregation-prone proteins (super aggregators) were obtained from Wallace et al. (Wallace et al. 2015). We used previously reported measures of protein abundance (Ho et al. 2018), protein–protein interactions (Michaelis et al. 2023), and dN/dS as a measure of protein divergence (Gonçalves et al. 2011). To include abundance as a covariate we used MaxQuant's estimates of protein abundance from the lowest temperature treatment. The average log2 abundance from the parental replicates was 6.48 for *S. cerevisiae* and 6.55 for *S. uvarum* and adding abundance data as a covariate had no significant effect on the average melting temperature difference between species using the linear model Tm ∼ species + abundance. Peptide detectability also had no association with the difference in melting temperatures between orthologs in all three experiments (Spearman's correlation, *P* > 0.05) and thus was not a confounding factor in our dataset.

### Protein Purification

#### Cloning

We cloned *S. cerevisiae* and *S. uvarum* alleles of *GUK1* and *AHA1* into a protein overexpression vector, AVA421 (Quartley et al. 2009) digested with *PmeI* and *NruI*, using NEBuilder HiFi DNA Assembly Cloning kit. This vector has an isopropyl ß-D-1-thiogalactopyranoside (IPTG) inducible promoter with an N terminal 6xHis tag. Assembled plasmids were transformed into DH5-alpha *E. coli* (New England Biolabs [NEB]) and sequenced. Confirmed plasmids were then transformed into BL21 (DE3) *E. coli* (NEB) for overexpression and purification.

#### Cell Harvest and Protein Purification

Three liters of lysogeny broth supplemented with 100 μg/mL of ampicillin was inoculated with 7 mL/L of overnight culture of BL21 (DE3) *E. coli* carrying an AVA421 plasmid with *GUK1* or *AHA1* from *S. cerevisiae* or *S. uvarum,* and grown at 30 °C until it reached an OD600 ∼0.5. Next, protein expression was induced using 1 mM IPTG, and the cultures were grown overnight at 18 °C. The next day, cells were spun down, washed with water, and quickly frozen on dry ice. The pellet was resuspended in sonication buffer (20 mM hydroxyethylpiperazine ethane sulfonic acid [HEPES] 7.5, 1 M NaCl, 5% glycerol, 2 mM beta-mercaptoethanol [BME], 2 μg/mL Leupeptin, 1 μg/mL Pepstatin, and 1 mM Pefabloc) and sonicated to lyse cells for 10 s for a total of eight cycles with 1 min of rest in between on ice.

Sonicated cells were spun down and the supernatant was diluted with an equal volume of buffer without salt (20 mM HEPES pH 7.5, 5% glycerol, 2 mM BME). The supernatant was applied to a 2-mL slurry of TALON Metal Affinity Resin (Takara Bio #635504) and nutated for 1 h to allow TALON to bind the His tagged protein. We then removed the supernatant and washed the resin four times with 25 mL 0.5 M NaCl wash buffer (20 mM HEPES pH 7.5, 5% glycerol, 0.5 M NaCl, 2 mM BME), nutating at 4 °C for 10 min. The sample was washed with 25 mL of E1 buffer (5 mM imidazole of pH 7.7 in 0.5 M wash buffer) followed by 25 mL of E2 buffer (10 mM imidazole of pH 7.7 in 0.5 M wash buffer) and applied to the TALON on a column. We eluted the protein by applying E3 buffer (250 mM imidazole of pH 7.7 in 0.5 M wash buffer) to the column.

Protein concentration was quantified using the Bradford assay, and 55 ul of Genscript 3C His protease was added for every 10 mg of protein to cleave the 6× His tag from the protein. The sample was dialyzed into 200 mM NaCl, 20 mM HEPES of pH 7.5, 5% glycerol, and 2 mM BME overnight. The next day, the protein was harvested, diluted with an equal volume of 0.8 M NaCl, 20 mM HEPES of pH 7.5, 5% glycerol, 2 mM BME and applied to a 1.5 mL slurry of TALON. We nutated the sample for 1 h at 4 °C and vacuum filtered the sample (0.2 μm). The sample was then dialyzed overnight into a storage buffer (20 mM HEPES of pH 7.5, 0.5 mM BME, and 10 mM NaCl). The following day, protein was harvested and frozen using liquid nitrogen for long-term storage at −80 °C.

#### Circular Dichroism Spectroscopy

We used circular dichroism (CD) spectroscopy to estimate the melting temperatures of the purified proteins. Guk1 proteins were diluted to a final concentration of 0.25 mg/mL in buffer (10 mM HEPES of pH 7.5, 0.25 mM BME, and 5 mM NaCl). We used a quartz cuvette (1-mm path length), and the buffer with no protein as a blank in a JASCO J-1100 Circular Dichroism spectrometer. We first performed a wavelength scan from 200 nm to 260 nm with 1-nm bandwidth and 0.1-nm step size to characterize the CD spectra of Guk1. Thermal melts were performed by tracking ellipticity at 222 nm for Guk1 from 4 °C to 74 °C with 1 °C/min increments with a 0.2 °C interval. Aggregation was irreversible when we reduced the temperature from 74 °C to 4 °C. Wavelength scans were also performed at seven temperatures during the thermal melt (supplementary fig. S4, Supplementary Material online). Three replicates of thermal melts were done for each Guk1 ortholog.

Aha1 was not stable in our buffer and crashed out when we thawed the protein. To avoid this interfering with CD, we spun the protein solution at 4 °C to remove insoluble protein and carried out thermal melts at 220 nm from 4 °C to 74 °C with 1 °C/min increments with a 0.2 °C interval. The wavelength scans were carried out from 210 nm to 260 nm with 1-nm bandwidth and 0.1-nm step size. Aggregation of Aha1 was also irreversible. Only one thermal melt was carried out for each of the two Aha1 orthologs.

We calculated mean residue molar ellipticity, by dividing the measured ellipticity (θ) by cuvette path length (l), protein concentration (c), and number of residues in the protein (N).


$$[\theta ]\cdot MR=\frac{100\cdot \theta }{l\cdot c\cdot N}$$

We scaled [*θ*]*_MR_* by its minimum value to obtain a metric of normalized secondary structure and then used the nls2 (v0.3–4) R package to fit a non-linear regression model to this metric and estimated the melting temperature. For Guk1, we calculated the average *[θ]_MR_* across the three replicates and then fit a model for a two-state transition from folded to unfolded states, allowing for linear changes in ellipticity pre- and post-transition (Model 2). For Aha1, we fit a model for a two-state transition from folded to unfolded states to the calculated normalized secondary structure (Model 1). Both models are from supplementary equations in Greenfield (Greenfield 2006). The estimated model parameters for all four proteins are in supplementary table S12, Supplementary Material online.

### Enzymatic Activity Measurements

#### Crude Protein Extraction and Temperature Treatments

*S. cerevisiae* (YJF155) and *S. uvarum* (YJF1449) cultures were grown in YPD to either mid-log (OD600 ∼ 0.6 to 0.8) or stationary phase (overnight) at 25 °C shaking at 300 rpm. Cells were washed twice with sterile water, and the pellets were stored at −80 °C until use. Frozen pellets were thawed and resuspended in 0.5 mL assay buffer (see assay descriptions) and transferred to Protein Lobind tubes (Eppendorf, Hamburg) containing 0.25-mL 0.5-mm glass beads. The lysates were pulverized in a Mini Bead-beater-96 (Biospec Products, Bartlesville, OK) over five cycles consisting of 90 s on ice followed by 30 s of homogenization. Lysates were clarified in a 4 °C centrifuge for 20 min at 18,000 *g*, and the protein concentration of the supernatants was determined by Pierce BCA assay (Thermo Fisher Scientific, Waltham, MA) using the assay buffers to dilute the samples and bovine serum albumin standards. Clarified samples were diluted to protein concentrations optimized for each assay, and 100-uL aliquots were transferred to PCR tubes for heat treatments ranging from 25 °C to 80 °C performed in thermocyclers (25 °C for 3 min, 3 min temperature treatment, and 25 °C for 3 min). Heated samples were transferred to Lobind tubes and clarified once again before loading into plates for enzymatic activity assays.

#### Malate Dehydrogenase Activity Assays

Malate dehydrogenase activity of soluble crude protein extracts was assessed using an assay kit (Sigma-Aldrich, St. Louis, MO). Clarified cell lysates were diluted to 8 µg/mL prior to heat treatment. Reaction mixes with or without malate were combined with heat-treated lysates in 96-well plates, and the absorbance at 450 nm was recorded every 2 min for an hour in a plate reader heated to 37 °C.

#### Glutathione Reductase Activity Assays

Glutathione reductase activity of soluble crude protein extracts harvested from stationary cultures was assessed using an assay kit (Sigma-Aldrich, St. Louis, MO) modified for use in a 96-well plate format. Clarified cell lysates were diluted to 500 µg/mL in the included assay dilution buffer prior to heat treatment. Assay reaction buffer (100 mM potassium phosphate buffered to pH 7.5, 1 mM EDTA, 150 uM reduced β-NADPH, 1 mM dithionitrobenzoic acid) with or without 1.3 mM oxidized glutathione was added to diluted samples in a 4:1 (vol:vol) ratio in 96 well plates. The absorbance at 412 nm was recorded every 10 s for 15 min in a plate reader.

#### Guanylate Kinase Activity Assays

Guanylate kinase activity of purified Guk1 from *S. cerevisiae* and *S. uvarum* was measured after exposure to various temperatures. Frozen proteins were resuspended in TPP buffer (20 mM sodium phosphate buffer, 50 mM sodium chloride, EDTA-free protease inhibitors, pH 7.4) and the concentration was determined by Pierce BCA assay. Protein concentrations were adjusted to 5 µg/mL with TPP buffer and subjected to heat treatments in a thermocycler as described above. Residual guanylate kinase activity was determined using the assay described by Lecoq et al (Lecoq et al. 2000) with adaptations made for a multi-well plate format. Assay buffer (100 mM Tris-HCl pH 7.5,100 mM KCl, 10 mM MgCl_2_, 300 uM reduced β-NADH (nicotinamide adenine dinucleotide reduced), 600 uM phosphoenolpyruvic acid, 2 mM ATP, 35 units pyruvate kinase from rabbit muscle, 80 units lactate dehydrogenase from rabbit muscle, and 880 uM GMP) was added to heat-treated samples in a 10:1 vol:vol ratio in 96 well plates. The absorbance at 340 nm was recorded every 10 s for 15 min in a plate reader heated to 30 °C. Guk1 kinetics were determined by combining non-heat-treated samples with the assay buffer without GMP in a 1:9 vol:vol ratio in a 96-well plate, pre-incubating the plate at temperature (30 °C or 37 °C) for 5 min, followed by initiating the assay with an equal sample volume of GMP for final concentrations ranging from 5 uM to 10 mM and measuring the absorbance at 340 nm every 10 s for 15 min in a plate reader heated to the pre-incubation temperature. We were unable to find conditions where Guk1 activity could be measured at high temperatures. Magnesium was previously identified to enhance the thermal stability of various proteins (Liu et al. 2007) in addition to increasing thermotolerance (Birch and Walker 2000; O’Connor et al. 2009), and we observed that it enhanced the residual enzymatic activity of Guk1 after exposure to higher temperatures when added to TPP buffer. However, magnesium is required by pyruvate kinase (Nowak and Suelter 1981) which produces pyruvate in proportion to the GDP produced by Guk1 and is used to generate the observable 340-nm absorbance change as β-NADH is converted to β-NAD by lactic dehydrogenase. We found that magnesium chloride concentrations above 2.5 mM caused measurable Guk1 thermoprotection while concentrations below 2.5 mM rendered pyruvate kinase to become the rate-limiting reaction in the assay.

#### Enzymatic Activity Assay Analyses

Enzymatic activity in a reaction was defined as the maximum change observed across 6 to 12 consecutive recorded time points. Activity rates for assays performed with crude protein extracts were corrected for intrinsic or off-target activity by subtracting maximum changes determined from substrate blank reactions performed with the same heat-treated sample and normalized to the rate observed for samples heat challenged at 25 °C. The inhibitory temperature at which 50% of the maximum observed activity remained was calculated using 5-point logistic regression via the nplr R package (Commo and Bot 2025). Statistical significance was tested using Students' two-tailed *t*-test.

### *GUK1* Allele Swap and Phenotyping

#### Allele Swap

We generated an allele replacement of *S. cerevisiae GUK1* with the *S. uvarum GUK1* in an *S. cerevisiae* background (YJF4559—Mat alpha hoΔ::dsdAMX4 ura3Δ derived from YPS163) under the native *S. cerevisiae* promoter. Since *GUK1* is essential, we used CRISPR to cut within *S. cerevisiae GUK1* (onw37 and onw38 in supplementary file S2, D, Supplementary Material online, see Laughery et Al. (Laughery et al. 2015) for cloning details) and provided a repair template consisting of *S. uvarum GUK1* (564 bp) flanked by ∼ 200 bp of *S. cerevisiae* upstream and downstream sequence to increase the chance of recombination occurring outside the *GUK1* coding sequence. This repair template was generated using NEB HiFi DNA assembly on pRS316 digested with BamHI and HindIII, three PCR purified fragments of ∼200 bp sequence upstream of *S. cerevisiae GUK1*, *S. uvarum GUK1* coding sequence and ∼200 bp sequence downstream of *S. cerevisiae GUK1*, and then amplified using external primers. We then screened transformants using allele-specific PCR (Chen and Schedl 2021) and Sanger sequenced Guk1 and flanking regions (primers listed in supplementary file S2, D, Supplementary Material online). Of 14 screened transformants, 9 were total gene replacements and 1 was a chimera (63.8% N terminal nucleotide sequence from *S. uvarum GUK1*). We used the same strategy to generate an allele replacement in *S. uvarum* (YJF4581) and obtained four full-length allele replacements and one chimera (83% N terminal sequence from *S. cerevisiae GUK1*) which were all confirmed by Sanger sequencing.

#### Phenotyping

*S. cerevisiae* (YJF4559), *S. cerevisiae* with *S. uvarum GUK1* (YJF5540), and *S. cerevisiae* with chimeric *GUK1* (YJF5548) were phenotyped at multiple temperatures 30 °C, 37 °C, 39 °C and 41 °C as well as at room temperature subsequent to a heat shock. For the constant temperature assays, strains were arrayed in a 96 colony format and a Singer Rotor HDA (Singer Instruments, Somerset, England) was used to pin the strains to agar plates made with synthetic complete media (CM), minimal media (MM) supplemented with uracil (50 mg/L), and minimal media supplemented with uracil and mycophenolic acid (MPA, 100 µg/mL), an inosine monophosphate dehydrogenase and guanosine biosynthesis inhibitor (Hyle et al. 2003). Plates were incubated at four temperatures for three days and colony size was measured using images obtained from a Singer Phenobooth (Singer Instruments). For the heat shock assays, the strains were arrayed on CM agar plates, incubated at 50 °C for 20, 40, 60, 100, 160, and 300 min, and then shifted to room temperature for an additional two days. Colonies on the edge of the plate as well as one row/column in were removed from the analysis to avoid edge effects (Miller et al. 2022). Colony size differences were tested using an analysis of variance with a linear model: size ∼ strain*temperature for the constant temperature and size ∼ strain separately for each heat shock duration. For allele replacements in *S. uvarum*, temperature-dependent growth differences were measured by spot dilutions on minimal medium plates supplemented with uracil at 25 °C, 30 °C, 31 °C, 32 °C, 33 °C, and 34 °C.

### Protein Stability Predictions

Protein structure predictions were generated using ColabFold (v1.5.2, https://github.com/YoshitakaMo/localcolabfold) (Mirdita et al. 2022). Protein sequences were obtained from complete and annotated genomes of *S. cerevisiae* (DBVPG6765) and *S. uvarum* (YJF1449 = CBS7001) (Fay et al. 2023). ColabFold was run without templates and with amber for structure refinement (relaxation/energy minimization). We obtained structures for both species orthologs for 5,097 out of 5,121 proteins.

Predicted changes in stability (ΔΔ*G*) were obtained for each *S. uvarum* amino acid substituted into the *S. cerevisiae* protein and vice versa. Proteins were aligned using Muscle (v3.8.31) (Edgar 2004) and amino acid substitutions were extracted from the alignments. We used both sequence and structure-based predictions from DDGun (https://github.com/biofold/ddgun) (Montanucci et al. 2019) and ACDC-NN (v0.0.17, https://github.com/compbiomed-unito/acdc-nn) (Benevenuta et al. 2021; Pancotti et al. 2021) since they have both been shown to have good antisymmetric properties in that forward and reverse changes are opposites of each other: ΔΔ*G*(*X* to *Y*) = −ΔΔ*G*(*Y* to *X*) (Pancotti et al. 2022). However, substitutions between species’ alleles may deviate from antisymmetry due to other amino acid differences between the two alleles. The correlation between the forward (*S. uvarum* amino acids into *S. cerevisiae* proteins) and reverse (opposite) changes was close to the negative one (supplementary file S2, E, Supplementary Material online). We thus used the average of the forward and negative of the reverse change so that a negative ΔΔ*G* indicates the *S. uvarum* amino acid is less stable than that of *S. cerevisiae*.

To control for uncertainty in predicted structures, we removed proteins with lower confidence structures and proteins with structures that differed from those originally produced by AlphaFold2 (Jumper et al. 2021). We used the per-residue confidence metric (pLDDT) from AlphaFold2 to remove 1,435 proteins with an average pLDDT across both species of less than 70. We used the lDDT metric from OpenStructure (v2.7.0) (Mariani et al. 2013) to compare our *S. cerevisiae* structures with those in the AlphaFold Database (Varadi et al. 2024) and removed 258 proteins with lDDT scores of less than 0.70. We used the sum of the ΔΔ*G* predictions for each protein as a measure of protein stability differences between species. In nearly all cases, proteins with higher confidence structures showed larger stability differences between species than proteins with lower confidence structures (supplementary table S9, Supplementary Material online). To determine whether stability differences are associated with sites within regions of low or high confidence structures we examined the sum of the ΔΔ*G* predictions across pLDDT categories using the filtered set of 3,646 proteins. For both sequence and structure-based methods, the majority of the stability differences came from high confidence (pLDDT > 80) sites (supplementary table S10, Supplementary Material online).

## Supplementary Material

msaf137_Supplementary_Data

## Data Availability

Raw data and data underlying the figures are available at OSF: https://osf.io/98c6n/. Raw proteomic data and the evidence files are available through the ProteomeXchange Consortium via the PRIDE repository with the identifier PXD058629.
