# Supplementary material for: Pervasive Divergence in Protein Thermostability is Mediated by Both Structural Changes and Cellular Environments
Source: Mol Biol Evol. 2025 Jun 6;42(7):msaf137. doi: 10.1093/molbev/msaf137 (PMC12227239; doi:10.1093/molbev/msaf137)
Supplement: msaf137_Supplementary_Data [file msaf137_supplementary_data.zip › Figures_supporting_revised.pdf]

E. HXK2 (YGL253W)

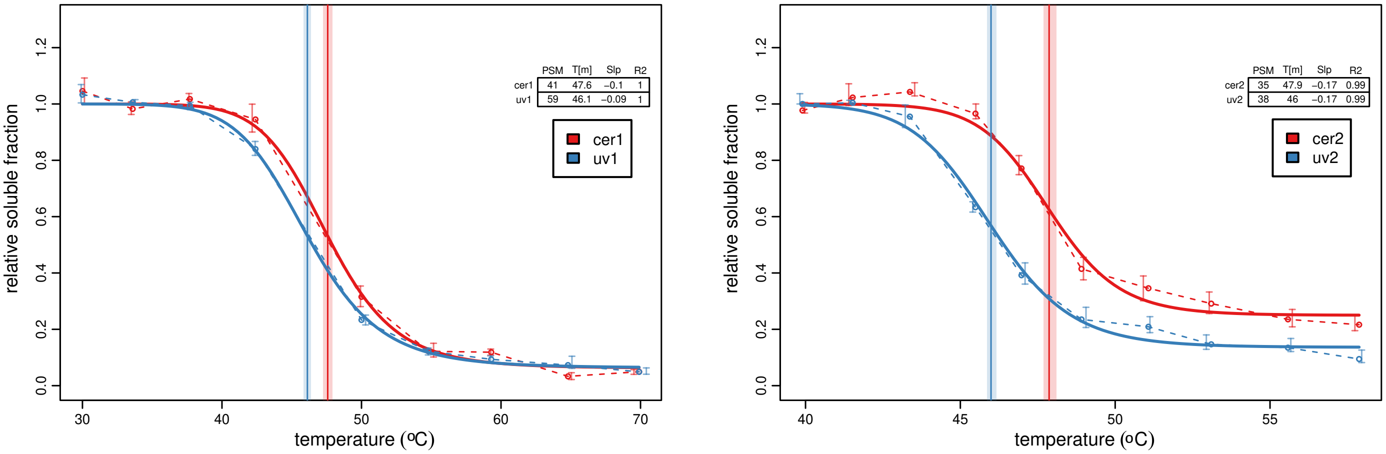

F. GLK1 (YCL040W)

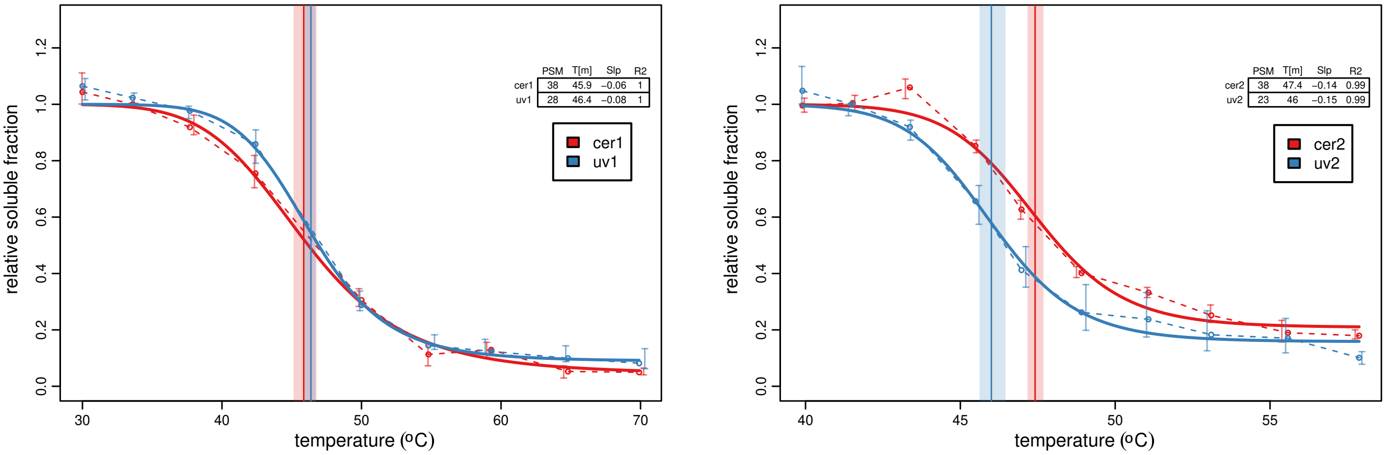

G. EMI2 (YDR516C)

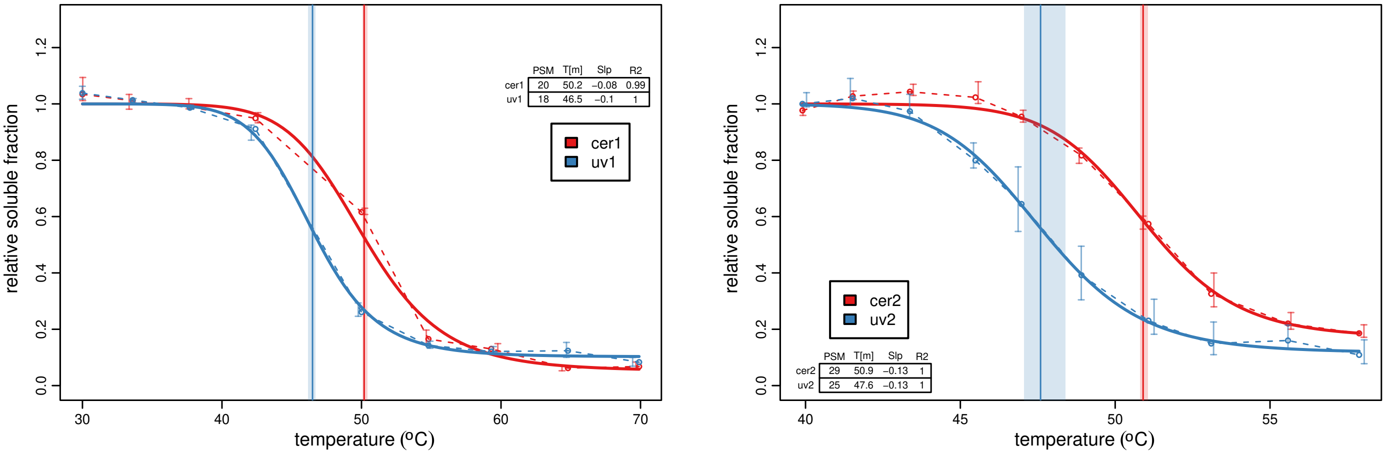

Figure S1: Solubility curves of seven proteins. Curves were fit using MSTherm to *S. cerevisiae* (red) and *S. uvarum* (blue) peptide spectra matches (PSMs) for parental replicates 1 (left) and 2 (right) as a function of temperature. Circles and whiskers show the mean PSM abundance and bootstrap confidence intervals. Vertical lines indicate estimated melting temperatures with shaded areas showing bootstrap confidence intervals. Inset shows the number of PSMs, melting temperature, slope and R^2 of each curve. Panels are MDH1 (A), MDH3 (B), GLR1 (C), HXK1 (D), HXK2 (E), GLK1 (F) and EMI2 (G).

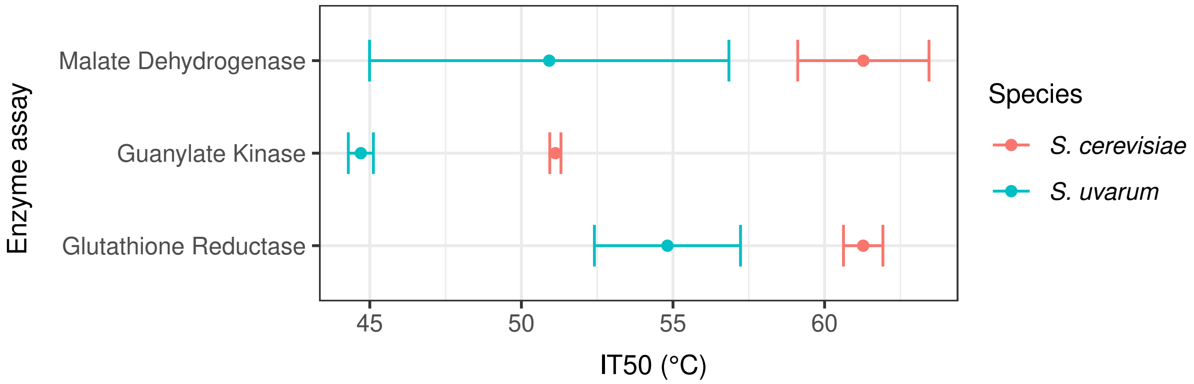

Figure S2: Thermal sensitivity of enzyme activity. *S. cerevisiae* and *S. uvarum* enzyme activity was measured from temperature treated lysates (malate dehydrogenase and glutathione reductase) or purified protein (guanylate kinase). The temperature at which activity was reduced 50% (IT50) is shown by the mean and its 95% confidence interval from three replicate measurements. For reference, the average of the parental species melting temperatures from the proteomics data was: 51.3 (Sc-Mdh1/3), 48.5 (Su-Mdh1/3), 50.8 (Sc-Guk1), 46.9 (Su-Guk1), 57.5 (Sc-Glr1), 49.9 (Su-Glr1).

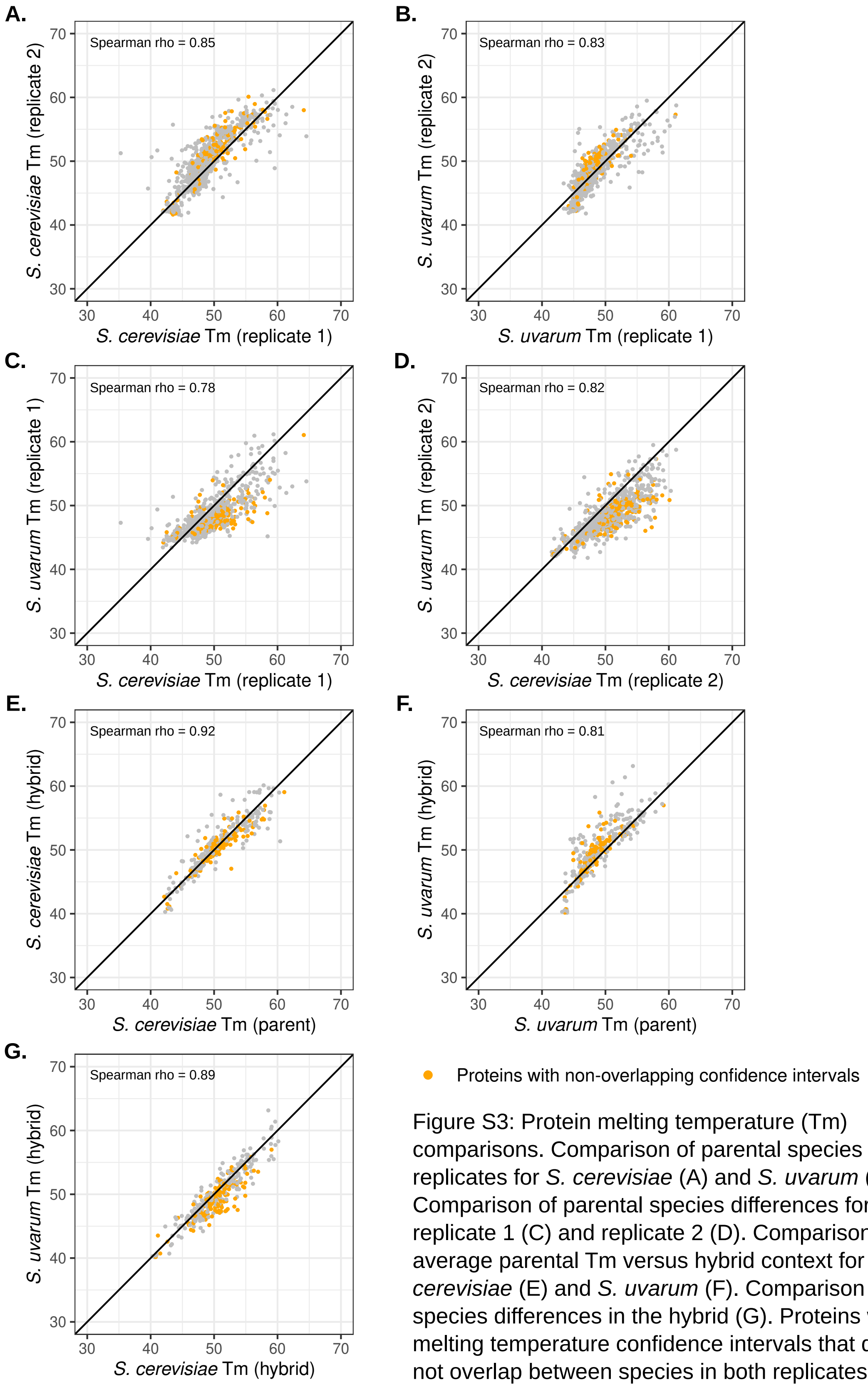

Figure S3: Protein melting temperature (Tm) comparisons. Comparison of parental species replicates for *S. cerevisiae* (A) and *S. uvarum* (B). Comparison of parental species differences for replicate 1 (C) and replicate 2 (D). Comparison of average parental Tm versus hybrid context for *S. cerevisiae* (E) and *S. uvarum* (F). Comparison of species differences in the hybrid (G). Proteins with melting temperature confidence intervals that did not overlap between species in both replicates are highlighted (orange).

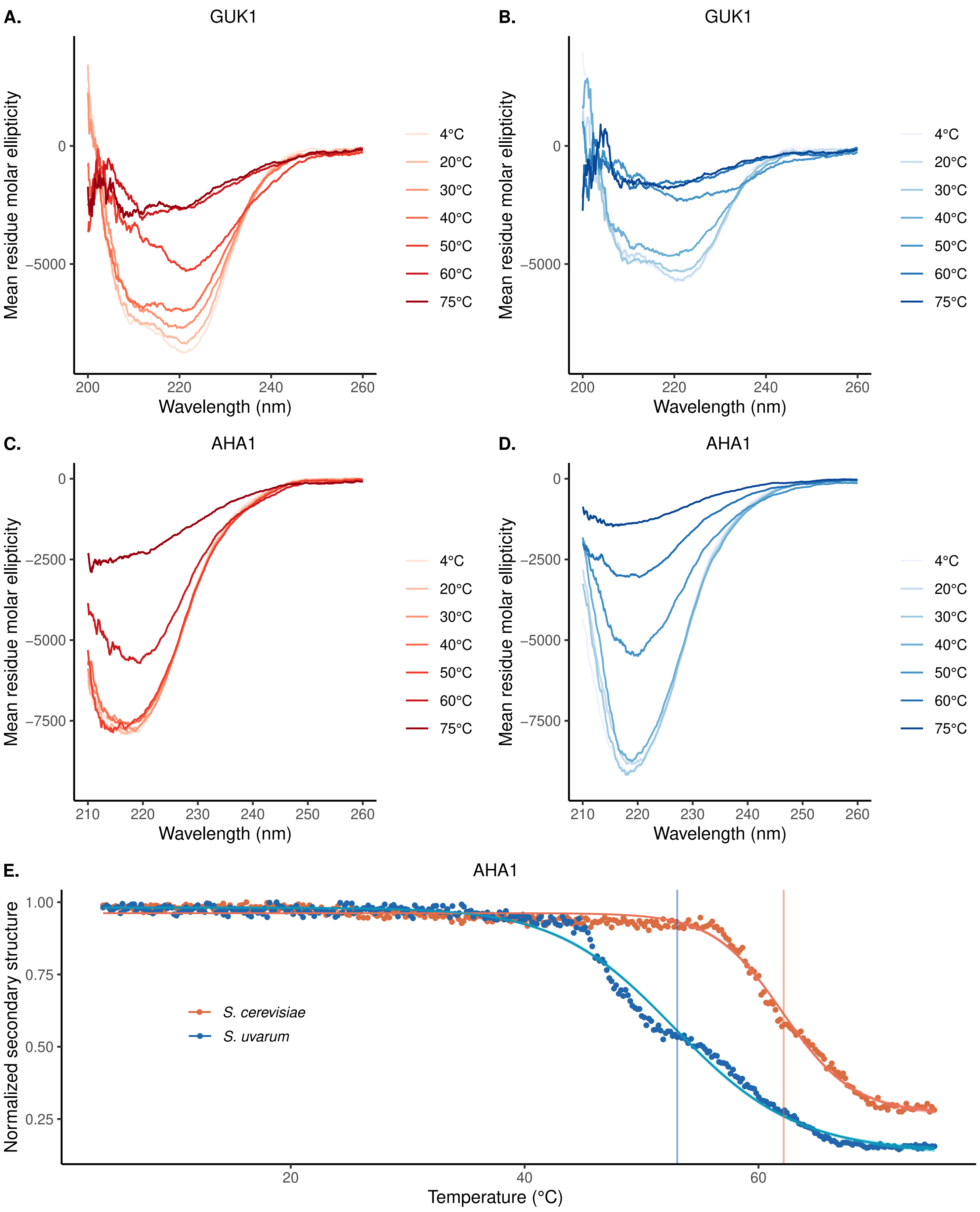

Figure S4: Circular dichroism wavelength scans at seven temperatures. Scans of Guk1 from *S. cerevisiae* (A) and *S. uvarum* (B), and Aha1 from *S. cerevisiae* (C) and *S. uvarum* (D) show changes in mean residue molar ellipticity (deg cm^2 $dmol^{-1}$ per residue) occur at higher temperatures. Loss of thermal stability of Aha1 is measured at 220 nm by normalized secondary structure which is mean residue molar ellipticity scaled by it's minimum value (E).

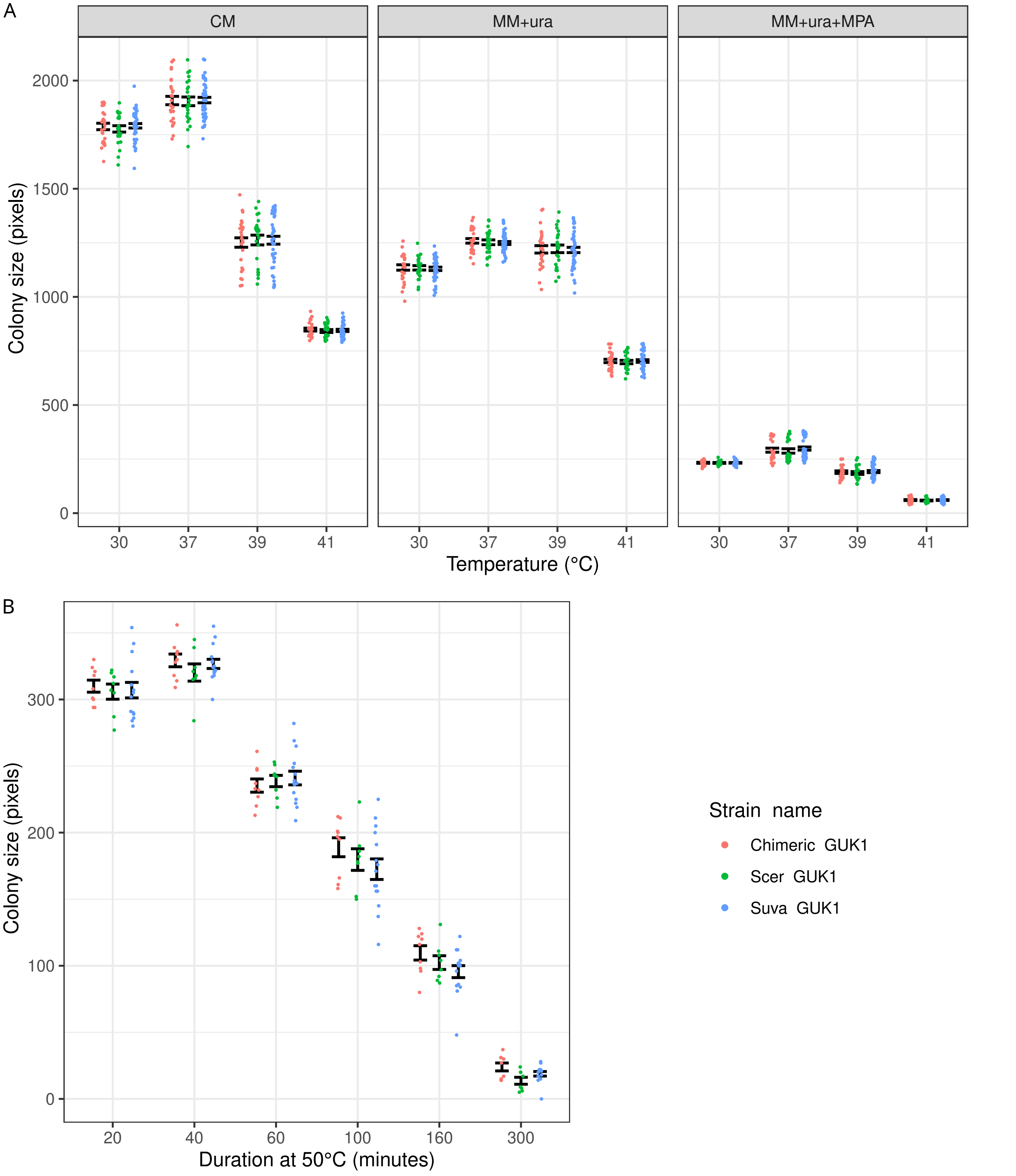

Figure S5: GUK1 allele-replacement phenotyping. (A) Colony size after three days of growth at different temperatures. Three panels show complete medium (CM), minimal media (MM) and minimal media with mycophenolic acid (MPA), an inhibitor of IMP dehydrogenase and synthesis of the guanosine monophosphate precursor XMP. (B) Colony size after two days of growth subsequent to a 50°C heat shock for different durations. Strains have an *S. cerevisiae* background with either *GUK1* from *S. uvarum* (Suva GUK1), *S. cerevisae* (Scer GUK1) or from a chimeric allele (Chimeric GUK1). Sample sizes are 8 (Scer), 9 (Chimera) and 15 (Suva) in panel A and 24 (Scer), 27 (Chimera), 45 (Suva) in panel B.

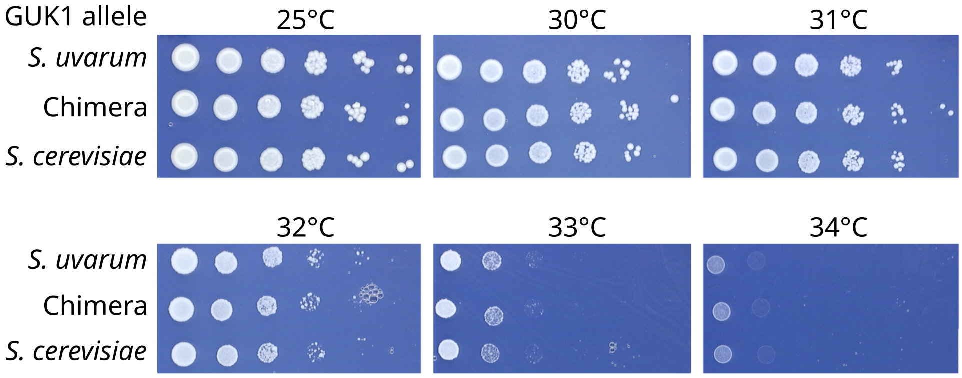

Figure S6: *GUK1* allele-replacement phenotyping in *S. uvarum*. *S. uvarum* strains with different *GUK1* alleles were diluted (left to right), spotted on complete medium and imaged after three days at different temperatures.
